# A Computational Exploration of the Interactions of the Green Tea Polyphenol (–)-Epigallocatechin 3-Gallate with Cardiac Muscle Troponin C

**DOI:** 10.1371/journal.pone.0070556

**Published:** 2013-07-29

**Authors:** Dominic Botten, Giorgia Fugallo, Franca Fraternali, Carla Molteni

**Affiliations:** 1 Physics Department, King's College London, London, United Kingdom; 2 Randall Division of Cell and Molecular Biophysics, King's College London, London, United Kingdom; German Research School for Simulation Science, Germany

## Abstract

Thanks to its polyphenols and phytochemicals, green tea is believed to have a number of health benefits, including protecting from heart disease, but its mechanism of action at the molecular level is still not understood. Here we explore, by means of atomistic simulations, how the most abundant of the green tea polyphenols, (–)-Epigallocatechin 3-Gallate (EGCg), interacts with the structural C terminal domain of cardiac muscle troponin C (cCTnC), a calcium binding protein that plays an important role in heart contractions. We find that EGCg favourably binds to the hydrophobic cleft of cCTnC consistently with solution NMR experiments. It also binds to cCTnC in the presence of the anchoring region of troponin I (cTnI(34–71)) at the interface between the E and H helices. This appears to affect the strength of the interaction between cCTnC and cTnI(34–71) and also counter-acts the effects of the Gly159Asp mutation, related to dilated cardiomyopathy. Our simulations support the picture that EGCg interacting with the C terminal domain of troponin C may help in regulating the calcium signalling either through competitive binding with the anchoring domain of cTnI or by affecting the interaction between cCTnC and cTnI(34–71).

## Introduction

**Figure 1:**
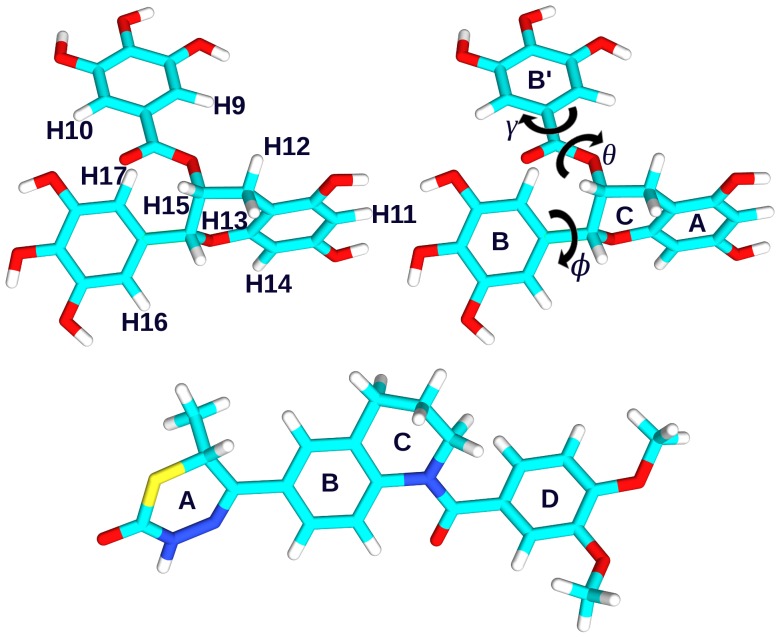
The structures of EGCg and EMD 57033. Top: the green tea polyphenol EGCg with labels for carbon bonded hydrogen atoms (left), and rings and dihedral angles (right). Bottom: the calcium sensitiser EMD 57033.

Tea, produced largely from the plant *Camellia Sinensis*, has been cultivated since ancient times and has been associated with Oriental medicine in the treatment of a variety of ailments. Green tea makes up nearly 20% of the total tea produced worldwide and is prepared in a way which prevents the oxidation of the leaf so that its polyphenolic content is preserved. Green tea polyphenols have been investigated in relation to a number of diseases, such as HIV, influenza, cancer, Alzheimer's, Parkinson's and cardiovascular diseases [1]–[11], with the final goal to ascertain whether the claimed health benefits can be demonstrated and understood and, if so, by which precise mechanism they are carried out. The most abundant of green tea polyphenols by weight is (–)-Epigallocatechin 3-Gallate (EGCg), which is shown in Fig. 1. It is a powerful antioxidant, which includes a benzenediol ring (label A) joined to a tetrahydropyran moiety (C), a pyrogallol ring (B) and a galloyl ring (B’). Rings B and B’ can assume a number of orientations with respect to A and C, depending on the torsional angles indicated as 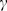, 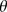 and 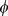 in Fig. 1.

Experimental studies have shown that green tea polyphenols, when interacting with the protein troponin C, found in cardiac muscles, may modulate the binding of Ca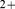 ions to the tissue. [12] The binding of calcium ions to cardiac tissue is essential for heart contraction and several diseases are known to modify such an interaction. Therefore the biochemical effects of these polyphenols are of great interest as they could potentially lead to prevention or new treatments of heart disease. [8], [13], [14].

Troponin is a protein which exists in both cardiac and skeletal muscles and has different functions depending on the location. Cardiac muscle troponin (cTn) plays a critical role in regulating relaxation and contraction in the cardiac thin filament. The protein is attached to tropomyosin and lies between actin filaments found in muscle tissue. It is composed of three distinct protein sub-units which have crucial roles in this regulatory function. Troponin C (cTnC) binds Ca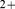 and regulates the thin filament activation, troponin I (cTnI) inhibits contraction in the absence of Ca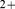 and troponin T (cTnT) attaches the troponin complex to tropomyosin on the actin filament. [15], [16] As shown by solution NMR [17], cTnC is a dumbbell shaped protein consisting of two domains, comprising the C and N terminals, connected by an elongated flexible linker. Each domain contains two EF-hand helix-loop-helix motifs which act to bind divalent cations, though in the N domain one of these sites is inactive. The hydrophobic core of the regulatory N terminal (cNTnC) domain is buried inside the protein, but unlike skeletal TnC, which exposes its hydrophobic core when bound to Ca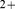, the regulatory domain is not thought to undergo major conformational changes upon binding Ca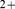. [17]–[21] The Ca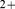 binding sites on the C terminal of TnC (cCTnC) are of high affinity and thought to remain bound throughout the cardiac cycle; without these ions bound the domain remains unstructured. [22].

cCTnC has been investigated as a possible target for drugs which modulate the calcium sensitivity of the protein (related to its propensity to bind Ca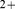 and pass this signal onto the rest of the protein complex). This is believed to be affected by mutations associated to dilated and hypertrophic cardiomyopathy. [12], [23], [24] Drugs in current use as treatment for heart diseases tend to alter the cytosolic calcium homeostasis and may provoke arythmia and even death. Hence there is a pressing need to find alternative therapies, such as calcium sensitisers and desensitisers. The structure of the calcium sensitiser EMD 57033, which we will also study for comparison, is shown in Fig. 1.

The C terminal domain of human cardiac troponin C in complex with EGCg has been studied by solution NMR. [13] A possible binding site was identified at the surface of cCTnC hydrophobic cleft. A three-dimensional structure for the cCTnC

**Figure 2:**
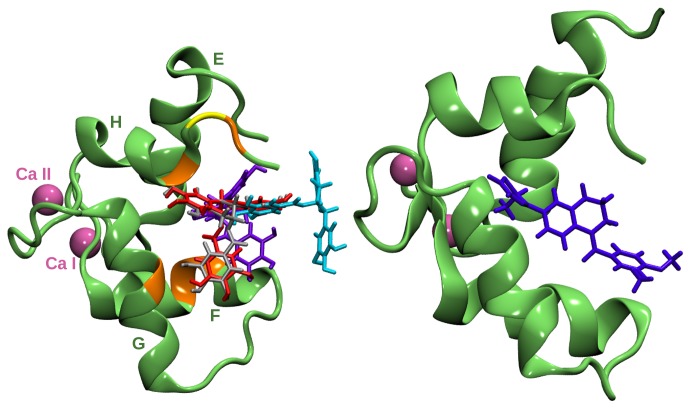
The C terminal domain of troponin C in complex with EGCg and EMD 57033. Left: cCTnC in complex with EGCg. Calcium ions are shown in mauve. The residues Met120, Leu121, Leu136, Met157 and Val160 are in orange, Gly159 in yellow. The solution NMR binding pose for EGCg (model M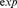) is in violet, [13] while the docked poses corresponding to models M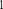, M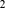 and M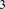 are respectively in grey, red and cyan. Right: cCTnC in complex with EMD 57033 [25] (model M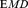).

2Ca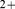 
EGCg complex has been proposed and is shown in Fig. 2; residues with significant nuclear Overhauser enhancement (NOE) contacts include Met120, Leu121, Leu136, Met157 and Val160 and are highlighted, together with Gly159 whose mutation to Asp is important for dilated cardiomyopathy. Mutations in the N terminal domain have been found to affect calcium sensitivity. [20] The binding site for EGCg in cCTnC is similar to that of the calcium sensitiser EMD 57033, which was also resolved by solution NMR spectroscopy [25] and is shown in Fig. 2. When the anchoring region of troponin I (cTnI(34–71)) was present (occupying the hydrophobic cleft), EGCg formed a ternary compound with cCTnC and cTnI [13]; no structure at atomic resolution of this ternary compound has been proposed based on spectroscopy experiments.

It is the aim of this study to provide an atomistic picture of the interactions of EGCg with the C terminal domain of cardiac muscle troponin C, which complements and most importantly expands the available experimental information. To achieve this goal, we used a series of computational techniques including ligand-protein docking, molecular dynamics (MD) and metadynamics [26], [27], a method for accelerating rare events and sampling free energy landscapes, to elucidate the mechanisms of binding of EGCg to cCTnC, both in the absence and presence of the anchoring domain of cTnI. Moreover, we analysed the EGCg influence on the Ca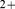 ions and assessed the effects of the mutation of Gly159Asp, which is related to dilated cardiomyopathy. We also compared the behaviour of EGCg with that of the calcium sensitiser EMD 57033 in complex with cCTnC. This extensive computational investigation provides a wealth of insights into the mechanisms of interaction and the dynamics of binding of EGCg with the C terminal domain of cardiac troponin C that may be useful for the development of new, complementary or preventative therapies for cardiovascular diseases.

## Methods

EGCg partial charges were evaluated so to reproduce the electrostatic potential (ESP) [28] in a density functional theory (DFT) calculation with the BLYP gradient corrected exchange and correlation functional [29], [30] and a 6–31++G** basis set, using the Gaussian03 package. [31].

Docking simulations were carried out with AUTODOCK [32] for EGCg docked into respectively cCTnC

2Ca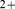 and cCTnC

2Ca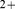 plus the anchoring domain of cTnI (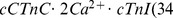
–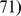); both these systems have been investigated experimentally in complex with EGCg. [13] The structure of cCTnC

2Ca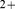 used for the docking was the lowest energy structure from the solution NMR experiment (PDB ID: 2KDH). [13] Preliminary blind docking studies including the entire protein were carried out, showing a clear preference for docking at the hydrophobic core. EGCg was then docked within a 94
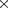
106
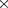
96 Å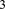 box, using a grid spacing set at 0.182 Å, which contained the hydrophobic cleft. The residues 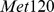, 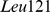, 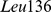, 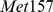 and 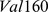, which were found experimentally to have significant NOE contacts with EGCg [13], as well as EGCg, were modelled as flexible. 200 Lamarckian genetic algorithm runs were carried out with a population size of 150 and set to carry out 25 million energy evaluations. The conformations were then clustered according to structural similarity using a 2 Å root mean square deviation criterion, and ranked according to binding energy. The lowest energy pose from the lowest energy and most populated cluster plus two other alternative poses were chosen as initial structures for MD simulations, as detailed in the following section, in addition to the experimental structure. [13] The cCTnC 2Ca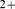 
cTnI(34–71) model was extracted from an X-ray crystal structure at 2.61 Å resolution of the core domain of human cardiac troponin in the Ca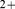 saturated form (PDB ID:1J1D), where cTnI(34–71) has an 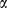-helical structure. [18], [33] As the binding site of EGCg to 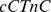  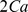 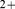  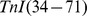 has not been experimentally resolved, a blind docking was undertaken covered the entire cCTnC portion of the protein complex with the same spacing as in the previous case; the lowest energy structure was selected as initial structure for MD.

For all molecular dynamics simulations the AMBER10 package [34], [35] was used with the AMBER ff03 force field. [36] The force-field parameters for EGCg were selected so to reproduce the torsional barriers about the dihedral angles 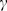, 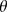 and 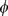 indicated in Fig. 1, as calculated with density functional theory. Standard amino acid protonation at neutral pH was considered; cCTnC contains many charged residues, in particular, besides two Arg and six Lys, eleven Glu and twelve Asp which provide a favourable environment for Ca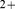 binding. Structures were neutralized with Na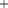 counterions (11 for cCTnC

2Ca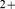 and 5 for 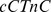  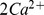  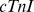
(34–71)), and explicitly solvated with a 12 Å buffer of TIP3P water molecules [37] in periodically repeated truncated octahedral cells. The SHAKE algorithm [38] was used to constrain the bonds including hydrogen atoms, allowing a time-step of 2 fs. A cutoff of 10 Å was used for the non-bonded interactions and electrostatic interactions were evaluated within a particle mesh Ewald scheme. Temperature was maintained at 300 K with a Langevin thermostat with a collision frequency of 1.0 ps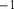 and pressure at 1 atm with a Berendsen barostat with a time constant of 2 ps. [39].

Structures were first minimized, then heated to room temperature. 50 ns NPT MD simulations were carried out at 300 K and 1 atm for all selected structures, of which the final 45 ns were used to calculate statistical averages. These include the average number of hydrogen bonds (defined as having a donor-acceptor distance smaller than 3.5 Å, or 4.0 Å when involving sulphur in Methionine, and a donor-H-acceptor angle larger than 120) and 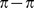 interactions (defined when aromatic rings are within a centre-to-centre distance of less than 5 Å of each other with the angle between the vectors normal to those rings smaller than 45°). Proton-proton distances were averaged over 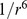 and equivalent atoms (with an upper cut off at 6 Å) in order to compare them with those derived from experimental NOE intensities.

For comparison, using a similar protocol, 50 ns MD simulations of cCTnC

2Ca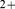 with no ligand and in complex with EMD 57033 [40] were also carried out. In the latter case the initial structure was the lowest energy structure from solution NMR spectroscopy (PDB ID: 1IH0). [25].

The free energies of binding were evaluated within the MM/PBSA method, using the 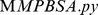 program within AMBER [41], which combines molecular mechanics energies with continuum solvation model approaches within the Poisson-Boltzmann Surface Area (PBSA) scheme. Trajectory snapshots used in the binding enthalpy calculations were taken every 10 ps of MD and a salt concentration of 0.1 mM was considered. A dielectric constant of 2.0 was used for the proteins, which contained several charged residues (e.g. 43% of cCTnC residues are charged), as this has been shown to improve the accuracy of the enthalpic contributions in such cases.[42]–[44] Entropic contributions were calculated using normal mode analysis on a sample of 20 snapshots. [41], [45], [46] There are a number of approximations in the MM/PBSA and normal mode scheme which limit its usefulness in predicting absolute free energy values; however meaningful trends can be obtained by comparing similar structures. [47], [48].

Metadynamics simulations [26], [27] were also performed in order to explore the free energy landscape of cCTnC

2Ca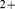 
EGCg as a function of selected relevant distances between the EGCg rings and cCTnC residues with significant experimental NOE contacts. The well-tempered scheme was chosen due to its efficient sampling speed and reduced error in free energy landscape reconstruction. [49] These calculations were undertaken using the PLUMED plug-in for AMBER10. [50] Specifically three distances between the average position of the methyl hydrogen atoms in the side chains of Met120, Leu136 and Val160 and the average positions of respectively EGCg H12/H13 on the A/C ring, H9/H10 on the B′ ring and H16/H17 on the B ring (see Fig. 1) were used as collective variables (CVs). These distances were selected as they are related to those inferred from the NOE intensities and will be indicated as CV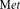, CV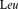 and CV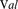. Gaussians of height equal to 0.1 kcal/mol and width 0.2 Å were deposited every ps and a bias factor of 10 was used. The sampled distances were limited by imposing a potential wall of magnitude 100 kcal/mol at 12 Å. All other computational details were as in the MD simulations. Free energies were averaged over 100 profiles. The starting configuration of cCTnC

2Ca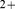 for the metadynamics simulation was chosen to be the solution NMR experimental structure equilibrated by MD.

## Results and Discussion

### cCTnC

2Ca
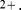
EGCg

We first investigated the interaction of EGCg with the C terminal domain of cTnC in order to provide details of the interaction mechanisms that extend the experimental solution NMR information. We carried out a series of molecular dynamics simulations for the cCTnC

2Ca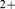 
EGCg complex starting from different initial configurations so to test whether EGCg can bind in different ways. One of the starting configurations for MD was the lowest energy experimental structure (M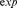). We also tested three docked structures as follows: (i) the lowest energy configuration from the most populated and lowest energy cluster (M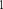), which is positioned towards the edge of the hydrophobic cleft; (ii) a configuration in the same cluster as M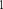 but roughly rotated by 180 with respect to the symmetry axis of the galloyl (B′) ring so that the positions of the A and B rings are swapped (M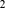); (iii) a configuration which does not dock directly to the hydrophobic cleft but slightly further out by the terminal strand (M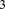) belonging to a cluster characterized by the third lowest energy and the second highest population. The initial positions of EGCg interacting with cCTnC are shown in Fig. 2. Additionally a model (M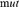) obtained by introducing the Gly159Asp mutation in M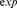 and a model with EMD 57033 (M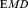) from solution NMR spectroscopy [25] were also studied. EMD 57033 binds more deeply inside the cleft than EGCg as shown in Fig. 2.

All models were stable as shown by the behaviour of the root mean square displacements (RMSD) of the backbone with respect to the optimized structure. In the 45 ns of simulation used for statistical analysis EGCg (and EMD 57033) remained bound in all models. The mutated cCTnC had relatively larger backbone fluctuations (on average 4.11 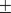 0.62 Å) than the wild type models (e.g. 3.09 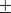 0.32 Å for M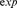), which were reduced to 3.92 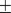 0.40 Å upon binding EGCg.

Hydrogen bonds formed by EGCg with the solvent and the protein were monitored throughout the MD simulations. EGCg alone in water showed significant hydrogen bonding to the surrounding solvent; e.g. in a 20 ns MD at ambient conditions of which the last 15 ns were used to calculate averages, EGCg formed a total of 18.8 hydrogen bonds with water molecules of which 11.1 with its oxygens as acceptors and 7.7 as donors. A small amount of intra-molecular hydrogen bonds (on average 0.8) on the EGCg B and B′ rings were detected, with very short individual lifetimes (less than 10% of the total simulation time) due to the competition with hydrogen bonds with the solvent; in fact, this amount increases to 1.7 in vacuo. The average number of hydrogen bonds EGCg formed with the C terminal domain of troponin C are listed in Table 1, together with residues involved in the bonds with large occupancies (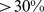). The total average number of hydrogen bonds formed by EGCg when in complex with cCTnC

2Ca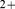 in water is reduced compared to the case in water alone, as expected by the proximity with the hydrophobic cleft. Residues Gly159 and Glu161 on the H helix are significant contributors to these interactions in all cases except M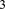 where EGCg attaches more closely to the G and F helices. The interaction with Gly159 may be important as evinced by the increased RMSD fluctuations noted for the simulations of the mutated system, which becomes heavily influenced by the binding of EGCg to the cleft. In the M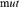 simulation EGCg is seen to shift slightly closer to the terminal strands of cCTnC enabling hydrogen bonding with both strands, effectively reducing fluctuations and stabilising the system. Interestingly it was not the mutated negatively charged residue at position 159, but its positively charged neighbour Lys158 to interact with EGCg through a hydrogen bond. With respect to EGCg, EMD 57033 has fewer atom groups capable of forming hydrogen bonds and bound deeply into the hydrophobic core of cCTnC leading to a substantially reduced overall amount of hydrogen bonds with both protein and solvent.

**Table 1:** Average numbers of hydrogen bonds formed by EGCg or EMD 57033 with the C terminal domain of troponin C.

Model	H 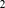 O	cCTnC	Total	cCTnC Residues
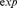 M	12.5	3.9	16.4	Met120, Gly159, Glu161
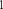 M	10.7	5.2	15.9	Phe156, Gly159, Glu161
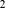 M	12.5	4.5	17.0	Glu126, Gly159, Glu161
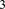 M	11.3	4.2	15.5	Thr124, Glu135, Asp139, Gly140
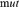 M	11.9	3.9	15.8	Gly91, Met120, Lys158
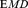 M	4.8	0.1	4.9	–

cCTnC residues which formed on average more than 0.3 hydrogen bonds with EGCg are listed.

cCTnC contains aromatic amino acids whose rings may participate in 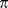-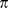 interactions with the aromatic rings of EGCg. Of the EGCg models, the only significant 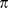-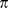 stacking behaviour was observed in M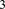 between EGCg and Phe156 which participated in 0.5 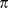
–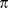-interactions on average. In M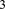 the looser binding of the ligand allowed the rings to more easily re-arrange into favourable stacking positions than in the other models. In the case for M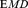, when monitoring the two EMD 57033 aromatic rings (B and D in Fig. 1) as well as the sulphur-containing ring (A), the average number of 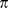-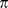 interactions was 1.2; these were essentially with Phe156, and to a much smaller degree (3.6%) with Phe104, with EMD 57033 rings A and B. The A ring was responsible for 69% of this interaction. The increased amount of 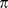-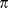 interactions compensated for fewer viable hydrogen bonds with the protein with respect to EGCg.

**Figure 3:**
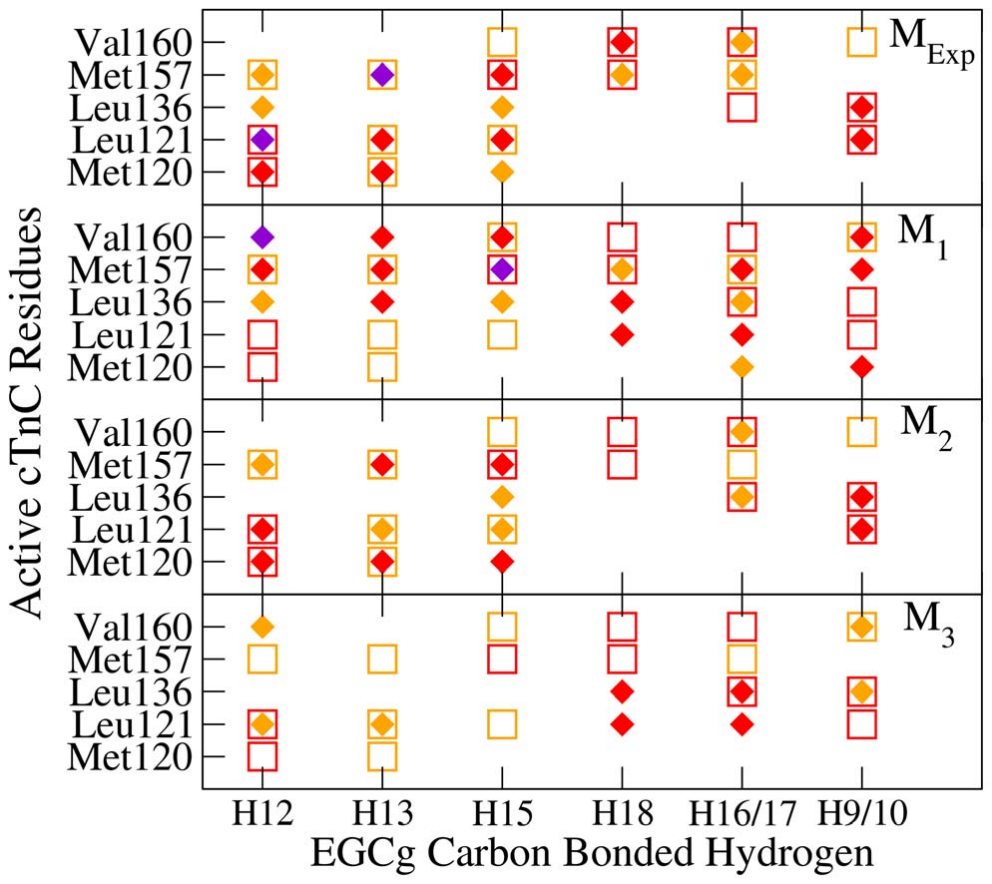
A comparison of calculated and experimental NOE distances. Calculated (diamond) and experimental [13] (square) NOE distances between select hydrogen atoms of EGCg (abscissa) and cCTnC residues (ordinate) in the cCTnC

The experimental NOE-related proton-proton distances [13] were compared with those evaluated in the MD simulations as shown in Fig. 3. The labels for the EGCg hydrogen atoms are the same used in the solution NMR reference [13] and are shown in Fig. 1. Equivalent atoms (H16 and H17 on ring B and H9 and H10 on ring B′) were averaged. Of 17 experimental NOE-related distances 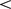
6 Å, 15 were also detected in the MD simulation of M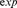, 8 in that of M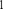, 12 in that of M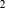 and only 5 in that of M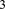. As expected M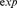 is in closest agreement with experiments followed by M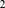, where EGCg has a similar initial ring orientation with respect to the protein cleft as M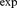. Despite an alternative docking orientation, M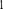 still shows a number of NOE-related distances consistent with experiments. M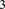 is the model that deviates the most from experiments, as expected from its binding at the edge of the hydrophobic cleft.

EGCg models. The colours correspond to the following ranges: medium (2.7 Å- 3.3 Å, purple); weak (3.3 Å- 5.0 Å, red); very weak (5.0 Å- 6.0 Å, orange).

**Figure 4:**
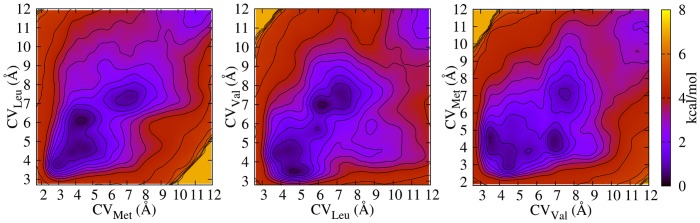
Free energy maps of the EGCg binding site in cCTnC. Projected (onto the third collective variable) free energy maps of cCTnC

Further exploration of the binding site was done using the metadynamics method for M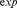 to map the free energy landscape as a function of selected collective variables which represent distances between groups of atoms of cCTnC and of EGCg related to significant NOE distances as described in the previous section. The three CVs map the distance between Val160 and the pyrogallol (B) ring (CV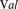), Met120 and the tetrahydrofuran (C) ring (CV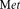) and Leu136 and the galloyl (B′) ring (CV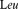). As apparent from the free energy maps in Fig. 4, there are several minima separated by small barriers at distances below 6 Å, plus some at larger distances, revealing that EGCg can easily readjust itself in various favourable slightly different positions with respect to representative residues at the hydrophobic cleft. These minima might act as a stepping points for EGCg to bind and unbind to the hydrophobic cleft, although this did not occur in the simulations due to the imposed potential walls.

2Ca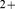 
EGCg as a function of the collective variables CV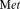, CV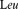 and CV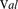 representing distances between groups of hydrogen atoms of EGCg and specific residues of cCTnC (i.e. Met120, Leu136 and Val160) linked to the measured NOE intensities, as described in the text. The free energy zero is set to the absolute minimum and the contour spacing to 0.5 kcal/mol.

Readjusting the orientation of the rings through torsions about the dihedral angles 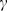, 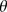 and 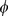 implies overcoming large energy barriers, except for 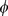 at specific values of 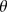 and 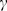. In fact, during the MD simulations, in all models 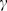 maintained the equivalent 0° or 180° values, 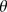 was mostly close to 90° with a small population at 150° for M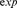, M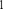 and M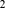, while it was mostly at 150° with a small population at 90° in M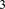. In M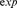, M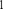 and M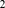, 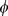 remained around 90° confirming that the pyrogallol ring did not substantially move in spite of relatively small torsional barriers due to the constraints imposed by the protein interactions. Conversely the pyrogallol ring of M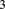 was exposed to the solvent and, therefore not feeling the interaction with the protein, could easily rotate.

**Figure 5:**
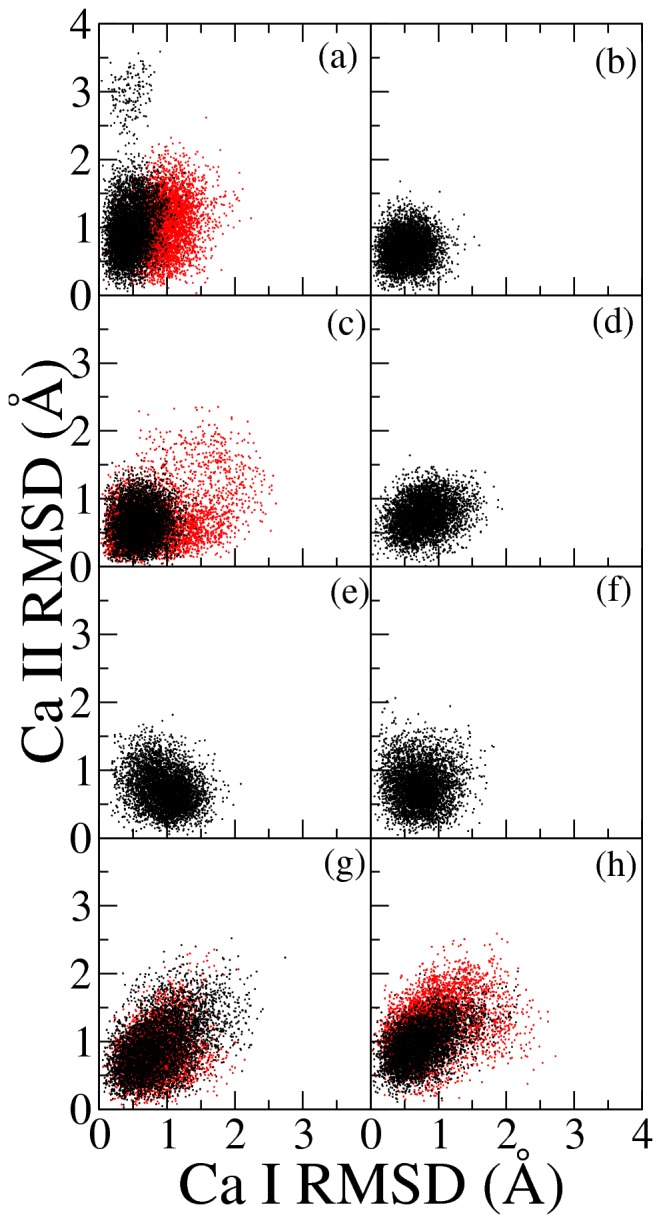
Mobility of the calcium ions. Distribution of the RMSD of Ca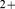 at site I versus site II for: (a) cCTnC with no ligand, (b) M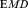, (c) M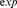, (d) M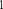, (e) M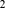, (f) M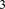, (g) cCTnC

The behaviours of the calcium ions at the two binding sites, which we labelled I and II in Fig. 2, were monitored and compared for the various models. While these sites are of high affinity and do not instigate the contraction-relaxation cycle in the same manner as the calcium binding to the N terminal domain, it has been shown that mutations which affect calcium sensitivity on the N terminal will also affect the C terminal sites [51] and thus the reverse may also hold true. The distributions of the RMSD of each calcium ion calculated with respect to their positions at the end of the MD equilibration for all the models aligned with respect to the cCTnC backbone are shown in Fig. 5. For cCTnC with no ligand, deformation to reduce solvent exposure of the hydrophobic core tended to confine the calcium ion at site I while having the opposite effect on site II. The destabilising effect on site II was so large that the corresponding calcium ion began a transition to a new location after nearly 48 ns (the simulation was extended a further 4 ns in order to map this transition). Interestingly, the mutated systems showed larger fluctuations overall, especially for the calcium ion at site I, though no transition to a different location was observed. This is consistent with the experimental observation that calcium ions show a reduction of binding affinities in cardiac tissue affected by dilated cardiomyopathy. [52]–[54] The calcium sensitiser EMD 57033 appears to highly restrict the movement of both calcium ions, which may provide a comparative example of sensitiser and/or regulator behaviour. The addition of EGCg binding to the hydrophobic cleft also markedly reduced fluctuations of both site I and II. Results for the cCTnC

2Ca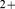 
TnI case will be discussed in the following section.

2Ca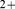 
TnI(34–71), (h) cCTnC

2Ca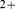 
TnI(34–71)

EGCg. Data related to the Gly159Asp mutation are shown in red.

The binding free energies of EGCg and EMD 57033 in the selected models, calculated within the MM/PBSA and normal mode analysis scheme, are shown in Table 2. The entropic contributions are fairly similar, as expected for related systems involving the same protein. The free energies should be considered relatively to each other rather than in absolute terms, as the enthalpic countribution depends on the chosen dielectric constant. In M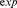, M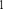 and M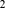 EGCg is similarly bound, with M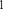 having the lowest average binding energy, showing that EGCg can bind in different poses with similar energetic contributions. However in M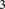 it is more loosely bound, as expected from the previous analysis. The average binding free energy in the presence of the Gly159Asp mutation is lower than that for wild type models. Experimental estimations from the dissociation constant for EGCg interacting with cCTnC depend on method and concentration [13], [55] and suggests a binding energy of at least −4.1 kcal/mol or lower [13]. EMD 57033 binds more strongly to cCTnC than EGCg consistently with a comparison of dissociation constants from NMR experiments. [13], [25].

**Table 2:** Enthalpic and entropic contributions and binding free energies between the complexes in square brackets evaluated within the MM/PBSA and normal mode analysis scheme.

Model	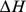 (kcal/mol)	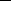 T 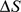 (kcal/mol)	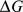 (kcal/mol)	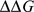 (kcal/mol)
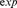 [cCTnC  2Ca 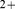 ]  [EGCg] - M	−27.6 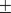 5.2	20.2 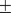 4.3	−7.4 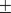 6.7	0.0
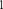 [cCTnC  2Ca 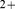 ]  [EGCg] - M	−27.7 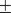 4.5	19.7 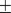 4.2	−8.1 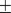 6.2	−0.7
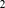 [cCTnC  2Ca 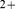 ]  [EGCg] - M	−25.0 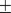 3.6	19.0 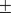 3.0	−5.9 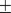 4.7	1.5
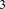 [cCTnC  2Ca 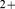 ]  [EGCg] - M	−21.9 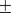 3.5	18.9 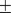 5.0	−3.1 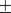 6.1	4.3
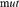 [cCTnC 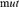  2Ca 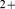 ]  [EGCg] - M	−28.1 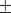 3.5	18.8 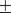 3.8	−9.4 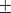 5.1	−2.0
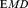 [cCTnC  2Ca 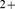 ]  [EMD 57033] - M	−31.1 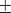 3.7	20.1 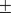 2.5	−11.0 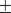 4.5	−3.6
[cCTnC  2Ca 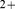  TnI]  [EGCg]	−9.4 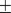 2.5	15.8 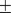 2.5	6.5 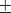 3.5	0.0
"	−11.6 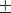 3.6	17.9 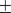 2.0	6.3 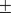 4.1	−0.2
[cCTnC 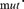 2Ca 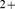  TnI]  [EGCg]	−14.0 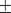 3.9	18.1 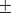 3.9	4.1 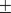 5.6	−2.4
"	−9.1 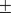 4.4	16.2 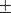 3.6	7.0 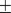 5.7	0.5
[cCTnC  2Ca 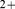 ]  [TnI]	−129.7 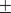 8.4	55.5 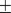 5.9	−74.2 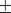 10.3	0.0
"	−130.1 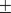 8.1	56.2 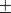 5.5	−73.9 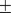 9.8	0.3
[cCTnC 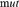  2Ca 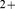 ]  [TnI]	−139.3 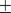 10.1	55.8 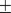 6.3	−83.5 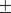 11.9	−9.3
"	−137.0 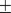 11.7	53.5 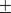 5.6	−83.4 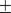 13.1	−9.2
[cCTnC  2Ca 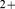  EGCg]  [TnI]	−121.0 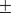 10.7	55.1 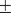 5.1	−65.8 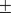 11.9	8.4
"	−123.1 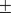 9.9	52.8 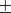 5.8	−70.3 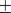 11.4	3.9
[cCTnC 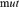  2Ca 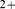  EGCg]  [TnI]	−126.4 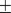 12.5	56.7 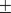 5.7	−69.7 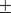 13.7	4.5
"	−121.6 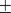 6.9	52.6 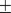 5.6	−70.0 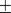 8.9	4.2

The top part of the table estimates the binding of EGCg or EMD 57033 to cCTnC; the middle part estimates the binding of EGCg when the anchoring part of cTnI is present; the bottom part of the table evaluates the inter-protein binding between the anchoring part of cTnI with cCTnC in the investigated models. Replica simulations are shown on consecutive rows.

### cCTnC

2Ca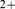 
TnI(34–71)

EGCg

We then investigated the interaction of EGCg with the C terminal of troponin C in the presence of the anchoring part of troponin I. We studied the cCTnC

2Ca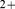 
TnI(34–71) complex in a manner similar to cCTnC through docking with EGCg followed by MD simulations. We considered models with and without EGCg, and with and without the Gly159Asp mutation. The goals of these calculations were to locate the binding site of EGCg when the hydrophobic cleft of cTnC is occupied by the anchoring part of cTnI, as well as monitor the effects of EGCg on the interaction between cCTnC and the cTnI anchoring peptide. Previous solution NMR experiments did not completely discern the binding site [13] in this case, though measured chemical shifts suggested likely binding areas. The effect of EGCg on the interaction between cCTnC and cTnI may influence the anchoring of cCTnC to the thin filament, affecting calcium sensitivity. Hence our simulations can provide very important details to elucidate the mechanisms of EGCg interaction which are still experimentally unavailable.

As the binding site was not known, a blind docking was undertaken using a grid which covered the entire cCTnC portion of the cCTnC

2Ca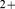 
TnI(34–71) complex. The docking results did not indicate a clear binding site as in the cCTnC

**Figure 6:**
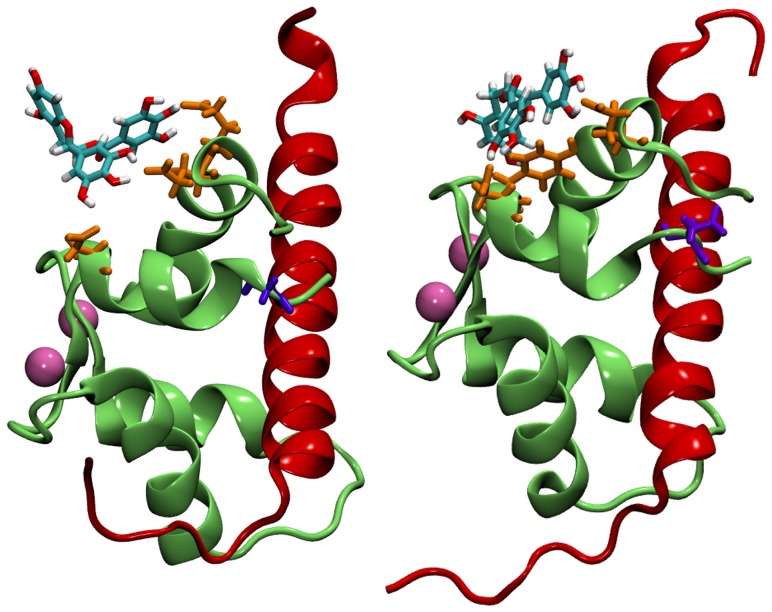
EGCg in complex with cCTnC when the anchoring region of cTnI is present. Molecular dynamics snapshots of cCTnC

2Ca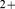 case, where the poses were highly clustered at low energy values. However, the poses found for EGCg were predominantly close to helix E or at the interface between helix E and H. The lowest energy pose that was chosen as the initial structure for MD simulations with EGCg, was initially interacting only with helix E, but within the equilibration time it reoriented at the interface between helix E and H and stayed there for the entire production run. A snapshot from MD simulations is shown in Fig. 6. With the cTnI fragment bound to its hydrophobic cleft, the structure of cCTnC was highly stable, as monitored through the backbone RMSD; cTnI(34–71) was also relatively stable although the terminal strands were very flexible and thus contributed significantly to the overall fluctuations of the system. To assess statistical effects that may affect the unstructured terminal strands, all MD simulations for the wild type and mutated cCTnC

2Ca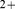 
TnI(34–71) complex were repeated twice; results for the replica simulations are reported in the relevant tables. The backbone root mean square fluctuations of the terminal strands of the cTnI fragment (defined as the 8 last residues on the C/N terminals respectively) were calculated over the production runs (compared against each replica average structure) and monitored for possible trends. When averaged over the eight residues and the eight MD simulations (with and without EGCg and with and without the Gly159Asp mutation), the C terminal strand of the cTnI fragment was seen to have fluctuations of 2.87 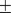 0.58 Å; the largest fluctuations for this terminal were observed in the cCTnC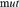 
2Ca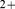 
TnI(34–71)

EGCg simulations, without which the average value reduced to 2.70
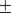
0.30 Å. The N terminal strand of the cTnI peptide had on average fluctuations of 2.68
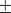
0.67 Å; in this case the fluctuations were larger when EGCg was present and for the mutated systems with respect to the wild type.

2Ca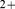 
TnI(34–71), wild type (left) and with the Gly159Asp mutation (right), interacting with EGCg. cCTnC is shown in green and cTnI(34–71) in red. The cCTnC residue at position 159 is in blue. The cCTnC residues which formed on average more than 0.4 hydrogen bonds with EGCg are in orange.

When interacting with cCTnC

2Ca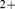 
TnI(34–71), EGCg formed on average 19.2 hydrogen bonds of which 4.4 were with cCTnC and 14.8 with the solvent. Protein-ligand hydrogen bonds were observed with the hydroxyl groups of the B and B′ rings, which considerably restricted the torsion of the 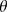 and 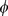 angles and acted to anchor the EGCg molecule to the E and H helices of cCTnC. Hydrogen bonds between EGCg and cCTnC were formed with the side-chains of the negatively charged residues Glu94 (on average 0.9) and Glu95 (2.3) on helix E as well as Asp151 (1.2) on helix H as shown in Fig. 6. In the presence of the Gly159Asp mutation, EGCg formed 16.7 hydrogen bonds, of which 13.3 with water and 3.4 with cCTnC, through a number of residues also including Glu94 (2.05) and Asp151 (0.41), besides Ser98 (0.24), Asp105 (0.12), Ala108 (0.11) and Tyr150 (0.44); a snapshot of this simulation is also shown in Fig. 6. 0.4 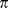-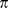 interactions were formed by EGCg with cCTnC

2Ca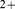 
TnI(34–71)

EGCg and 0.5 with cCTnC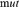 
2Ca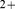 
TnI(34–71)

EGCg, in both cases with the Tyr150 on helix H.

It was shown by NMR spectroscopy that EGCg forms a ternary compound with cCTnC

2Ca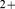 
TnI(34–71), although decreased chemical shift perturbations and lower affinity with respect to the case when the cTnI anchoring fragment was absent were observed [13]. This is at variance from EMD 50733, which does not bind concurrently with cTnI(34–71), but does bind when the inhibitory domain cTnI(128–147) is present [25]: hence it was argued that EMD 50733 action as a calcium sensitizer may be related to the weakening of the interaction with cTnI(34–71) through competitive binding with the hydrophobic cleft, increasing the propensity of binding the inhibitory domain cTnI(128–147) to cCTnC

2Ca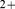 [13], [25]. In the presence of EGCg, the largest chemical shifts for cCTnC were recorded close to the E-H helix interface, along the F helix and the E-F loop, although discriminating between direct contact with EGCg and conformational changes due to EGCg binding to the complex cCTnC

2Ca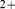 
TnI(34–71) was not possible. [13] In ref. [13] the binding site was hypothesized to be close to the F helix; however our simulations would suggest a binding site at the E-H interface, which is still consistent with the experimental chemical shifts at this site. EGCg may also be able to bind in more than one location when the cCTnC hydrophobic cleft is occupied by the anchoring section of cTnI.

The relative binding free energy in Table 2 shows a weakening in the binding of EGCg, with respect to the models discussed in the previous section: in fact EGCg interacts more loosely with cCTnC when the hydrophobic cleft, i.e. the obvious binding cavity, is occupied by the cTnI anchoring peptide, consistently with the docking results and as observed in the experiments [13]. When entropic contributions are included, the binding free energy becomes slightly positive; however this energy should not considered as an absolute value and EGCg remained bound to cCTnC

2Ca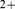 
TnI(34–71) for the entire length of the MD simulations, both in the wild type and mutated systems.

The root mean square displacements of the cCTnC bound calcium ions were monitored throughout the MD simulations and are shown in Fig. 5. In the simulations including cTnI with and without EGCg, the ions appear less restrained than previously observed without the cTnI peptide. As the cTnI fragment occupied the cCTnC hydrophobic cleft, the variation in position of each calcium ion was similar with respect to each-other: the occupation of the cleft, by a ligand or protein fragment, seemed to prevent significant structural changes of the calcium binding sites affecting the calcium binding. The presence of EGCg did not seem to strongly affect the calcium RMSD in the wild type system, while it slightly increased them in the mutated system.

**Figure 7:**
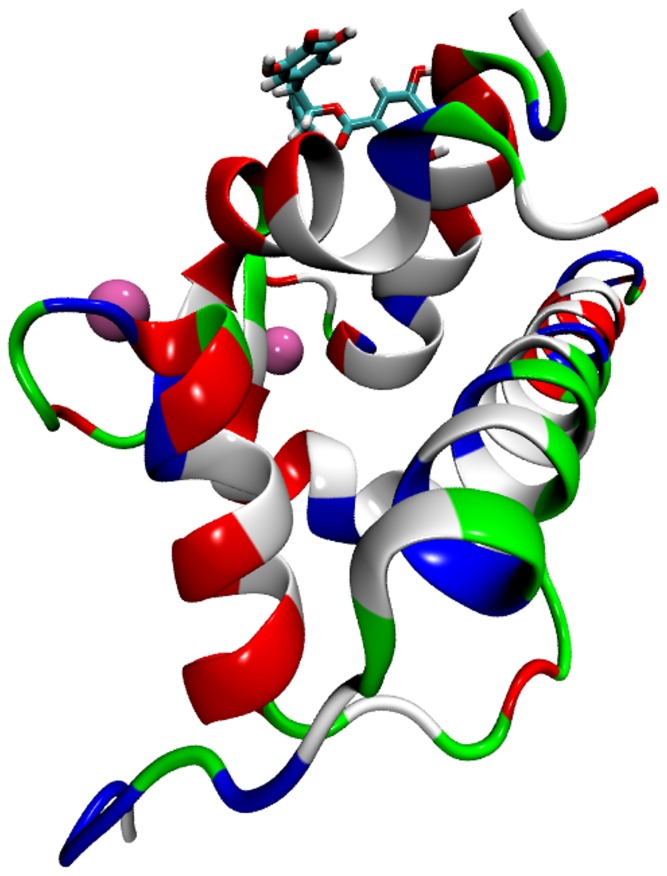
Distribution of charged amino acid in cCTnC

We then analyzed the interaction between cCTnC and cTnI(34–71) in the absence and presence of EGCg. As shown in Table 3, cCTnC and cTnI(34–71) interacted via several hydrogen bonds most of which are salt bridges between pair of amino acids of opposite charge as evident from Fig. 7. The observed dominance of salt bridges is consistent with the experimental suggestion that cTnC-cTnI binding is electrostatically driven [56]. The total average number of hydrogen bonds was increased with the mutation of Gly159 with the negativly charged Asp, as we would intuitively expect. However the increased number of hydrogen bonds in the mutated systems, which lead to stronger binding, seems to be in contrast with NMR experiments which detected a slight weakening. [24] Overall, the presence of EGCg reduced or maintained the average number of hydrogen bonds, which may be related to a weakening of the interaction between cCTnC and cTnI(34–71).

2Ca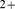 
TnI(34-71). Molecular dynamics snapshot of cCTnC

2Ca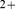 
TnI(34–71) interacting with EGCg coloured according to residue type: non-polar (white), polar (green), positively charged (blue) and negatively charged (red).

**Table 3:** Average number of the inter-protein hydrogen bonds and salt bridges between cCTnC

2Ca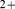 and cTnI(34-71) (wild type and with the Gly159Asp mutation) in the presence and absence of EGCg.

Model	Hydrogen Bonds	Salt Bridges
[cCTnC  2Ca 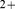 ]  [TnI]	11.2	8.8 (78 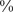 )
"	10.5	7.8 (74 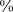 )
[cCTnC 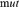  2Ca 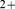 ]  [TnI]	15.9	11.5 (72 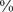 )
"	15.6	11.3 (72 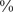 )
[cCTnC  2Ca 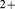  EGCg]  [TnI]	10.6	8.0 (75 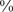 )
"	10.6	8.5 (80 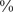 )
[cCTnC 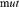  2Ca 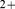  EGCg]  [TnI]	12.5	8.6 (69 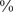 )
"	12.5	9.5 (76 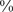 )

The percentage of salt bridges with respect to the total number of hydrogen bonds is shown in brackets. Replica simulations are shown on consecutive rows.

The behaviour of the hydrogen bonds is mirrored by the trend observed in the free energies of binding between cCTnC and cTnI(34–71), shown in Table 2: the effect of the Gly159Asp mutation is to strengthen the inter-protein binding while the interaction with EGCg tends to weaken such an interaction. Differences in the average values of the cTnI fragment binding free energy in the cCTnC

2Ca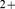 
TnI(34–71)

EGCg replicas may be attributed to the cTnI N terminal strand fluctuations; the calculated values are however within the statistical error. The low values of the binding enthalpies are due to the flexible cTnI N terminal strand interacting with cCTnC and in fact can be reduced by cutting this tail and performing the calculations for a simplified model, without modifying the trend.

### Conclusions

In this work the binding site of the green tea polyphenol EGCg in the C terminal domain of cardiac muscle troponin C was computationally confirmed to be in the proximity of the hydrophobic cleft, consistently with solution NMR data [13]: we found that EGCg can bind in different orientations with fairly similar energies. The molecular interactions which occured between EGCg and the cardiac protein were thoroughly analyzed. The two calcium ions bound to cCTnC behaved similarly to each other when either EGCg or EMD 57033 bind to cCTnC, although the latter, known as a calcium sensitiser, binds more strongly and deeply into the hydrophobic cleft. The relatively rigid A/C and B′ rings of EGCg tended to orient themselves so to minimise the hydrophobic cleft exposure to the solvent, leaving the B ring able to rotate flexibly. The hydroxyl groups attached to the EGCg aromatic rings were then able to favourably form hydrogen bonds with residues at the edge of the cleft.

Moreover, EGCg was shown to bind, although less strongly, to the cCTnC

2Ca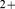 
TnI(34–71) complex at the interface between the E and H helices. In this position EGCg appeared to weaken the binding affinity of cCTnC for cTnI(34–71), which would then modulate the anchoring of the cTnC protein, changing the force generation response.

When the Gly159Asp mutation was present, in all systems but cCTnC

2Ca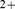 
TnI(34–71) the high affinity calcium sites showed on average larger displacements than in the corresponding wild type systems, and this effect may be exacerbated for the N terminal calcium site as this is already of low affinity. The Gly159Asp mutation strengthened the interaction, mostly mediated by salt bridges between cCTnC and cTnI(34–71) so the presence of EGCg may compensate this effect. Further investigations on other known cardiac mutations would be important to ascertain whether a cooperative effect on calcium affinity and thin filament binding occurs.

Although the mechanisms of calcium sensitisation and desensitisation are very complex functions of subsequent events and hence difficult to unravel, these results suggest that EGCg may have a “regulating” effect on the troponin system to which it can bind both in the absence (or in competition) and presence of cTnI, affecting in the latter case the strength of the protein-protein interaction and hence the calcium signalling transmission. Hence EGCg is an interesting candidate to be considered as a basis for further drug development or simply as a preventitive aid against cardiovascular disease. In addition, the E-H helix interface of cCTnC should be considered for further studies as, even if binding with low affinity, EGCg was able to affect the inter-protein interaction and drugs which can effectively target this region may prove invaluable in treatments of heart disease; alternative binding locations could also be explored.

## References

[1] Hamza A, Zhan C (2006) How can (–)-epigallocatechin gallate from green tea prevent HIV-1 infection? J Phys Chem B 110: 2910–2917. 16471901 10.1021/jp0550762

[2] Yamaguci A, Honda M, Ikigai H, Hara Y, Shimamura T (2002) Inhibitory effects of (–)-epigallocatechin gallate on the life cycle of human immunodeficiency virus type 1 (HIV-1). Antiviral Research 53: 19–34. 11684313 10.1016/s0166-3542(01)00189-9

[3] Nakayama M, Suzuki K, Toda M, Okubo S, Hara Y, et al (1993) Inhibition of the infectivity of inuenza virus by tea polyphenols. Antiviral Research 21: 289–299. 8215301 10.1016/0166-3542(93)90008-7

[4] Ehrnhoefer DE, Bieschke J, Boeddrich A, Herbst M, Masino L, et al (2008) EGCg redirects amyloidogenic polypeptides into unstructured, off-pathway oligomers. Nat Struct Mol Biol 15: 558–566. 18511942 10.1038/nsmb.1437

[5] Bieschke J, Russ J, Friedrich RP, Ehrnhoefer DE, Wobst H, et al (2010) EGCg remodels mature *α*-synuclein and amyloid-*β* fibrils and reduces cellular toxicity. Proc Natl Acad Sci U S A 107: 7710–7715. 20385841 10.1073/pnas.0910723107 PMC2867908

[6] Hertog M, Feskens E, Hollman P, Katan M, Kromhout D (1992) Dietary antioxidant avonoids and risk of coronary heart disease: the zutphen elderly study. Lancet 339: 1523–1526. 8105262 10.1016/0140-6736(93)92876-u

[7] Riemersma R, Rice-Evans C, Tyrrell R, Clifford M, Lean M (2001) Tea avonoids and cardiovascular health. Q J Med 94: 277. 10.1093/qjmed/94.5.277 11353103

[8] Hodgson M (2008) Tea avonoids and cardiovascular disease. Asia Pac J Clin Nutr 17: 288–290. 18296358

[9] Rathore K, Choudhary S, Odoi A, Wang HCR (2012) Green tea catechin intervention of reactive oxygen species-mediated ERK pathway activation and chronically induced breast cell carcinogenesis. J Carcinog 33: 174–183. 10.1093/carcin/bgr244 PMC3276334 22045026

[10] Kostin SF, McDonald DE, McFadden DW (2012) Inhibitory effects of (–)-epigallocatechin-3-gallate and pterostilbene on pancreatic cancer growth in vitro. J Surg Res 177: 255–262. 22583593 10.1016/j.jss.2012.04.023

[11] Singh BN, Shankar S, Srivastava RK (2011) Green tea catechin, epigallocatechin-3-gallate (EGCg): Mechanisms, perspectives and clinical applications. Biochem Pharm 82: 1807–1821. 21827739 10.1016/j.bcp.2011.07.093 PMC4082721

[12] Tadano N, Du C, Yumoto F, Morimoto S, Ohta M, et al (2010) Biological actions of green tea catechins on cardiac troponin C. Br J Pharmacol. 161: 1034–1043. 10.1111/j.1476-5381.2010.00942.x PMC2998685 20977454

[13] Robertson I, Li M, Sykes B (2009) Solution structure of human cardiac troponin C in complex with the green tea polyphenol, (–)-epigallocatechin 3-gallate. J Biol Chem 284: 23012–23022. 19542563 10.1074/jbc.M109.021352 PMC2755708

[14] Wong WW, Gerson JH, Rubenstein PA, Reisler E (2002) Thin filament regulation and ionic interactions between the N-terminal region in actin and troponin. Biophys J 83: 2726–2732. 12414705 10.1016/S0006-3495(02)75282-X PMC1302357

[15] Parmacek MS, Solaro RJ (2004) Biology of the troponin complex in cardiac myocytes. Prog Cardiovasc Dis 47: 159–176. 15736582 10.1016/j.pcad.2004.07.003

[16] Gomes AV, Potter JD, Szczesna-Cordary D (2002) The role of troponins in muscle contraction. IUBMB Life 54: 323–333. 12665242 10.1080/15216540216037

[17] Sia S, Li M, Spyracopoulos L, Gagne S, Liu W, et al (1997) Structure of cardiac muscle troponin C unexpectedly reveals a closed regulatory domain. J Biol Chem 272: 18216–18221. 9218458 10.1074/jbc.272.29.18216

[18] Takeda S, Yamashita A, Maeda K, Maeda Y (2003) Structure of the core domain of human cardiac troponin in the Ca2+ saturated form. Nature 424: 35–41. 12840750 10.1038/nature01780

[19] Spyracopoulos L, Li M, Sia S, Gagne S, Chandra M, et al (1997) Calcium-induced structural transition in the regulatory domain of human cardiac troponin C. Biochemistry. 36: 12138–12146. 10.1021/bi971223d 9315850

[20] Kekenes-Huskey PM, Lindert S, McCammon JA (2012) Molecular basis of calcium-sensitizing and desensitizing mutations of the human cardiac troponin c regulatory domain: A multi-scale simulation study. PLoS Computational Biology 8: e1002777. 23209387 10.1371/journal.pcbi.1002777 PMC3510055

[21] Lindert S, Kekenes-Huskey PM, Huber G, Pierce L, McCammon JA (2012) Dynamics and Calcium Association to the N-Terminal Regulatory Domain of Human Cardiac Troponin C: A Multiscale Computational Study. J Phys Chem B 116: 8449–8459. 22329450 10.1021/jp212173f PMC3405770

[22] Mercier P, Li M, Sykes B (2000) Role of the structural domain of troponin C in muscle regulation: NMR studies of Ca2+ binding and subsequent interactions with regions 1–40 and 96–115 of troponin I. Biochemistry. 39: 2902–2911. 10.1021/bi992579n 10715110

[23] Yanaga F, Morimoto S, Ohtsuki I (1999) Ca2+ sensitization and potentiation of the maximum level of myofibrillar atpase activity caused by mutations of troponin T found in familial hypertrophic cardiomyopathy. J Biol Chem 274: 8806–8812. 10085122 10.1074/jbc.274.13.8806

[24] Baryshnikova OK, Robertson IM, Mercier P, Sykes BD (2008) The dilated cardiomyopathy G159D mutation in cardiac troponin C weakens the anchoring interaction with troponin I. Biochemistry. 47: 10950–10960. 10.1021/bi801165c 18803402

[25] Li M, Spyracopoulos L, Beier N, Putkey J, Sykes B (2000) Interaction of cardiac troponin C with Ca2+ sensitizer EMD 57033 and cardiac troponin I inhibitory peptide. Biochemistry 39: 8782–8790. 10913289 10.1021/bi000473i

[26] Laio A, Parrinello M (2002) Escaping free energy minima. Proc Natl Acad Sci U S A 99: 12562–12566. 12271136 10.1073/pnas.202427399 PMC130499

[27] Laio A, Gervasio FL (2008) Metadynamics: a method to simulate rare events and reconstruct the free energy in biophysics, chemistry and material science. Rep Prog Phys 71: 126601.

[28] Singh U, Kollman P (1984) An approach to computing electrostatic charges for molecules. J Comput Chem 5: 129–145.

[29] Becke AD (1988) Density-functional exchange-energy approximation with correct asymptotic-behavior. Phys Rev A 38: 3098–3100. 10.1103/physreva.38.3098 9900728

[30] Lee C, Yang W, Parr RG (1988) Development of the Colle-Salvetti correlation-energy formula into a functional of the electron density. Phys Rev B 37: 785–789. 10.1103/physrevb.37.785 9944570

[31] Frisch MJ, Trucks GW, Schlegel HB, Scuseria GE, Robb MA, et al.. (2004) Gaussian 03 revision c.02. Technical report, Gaussian, Inc., Wallingford, CT.

[32] Morris G, Goodsell D, Halliday R, Huey R, Hart W, et al (1998) Automated docking using a lamarckian genetic algorithm and an empirical binding free energy function. J Comput Chem 19: 1639–1662.

[33] Berman HM, Westbrook J, Feng Z, Gilliland G, Bhat TN, et al (2000) The protein data bank. Nucleic Acids Res 28: 235–242. 10592235 10.1093/nar/28.1.235 PMC102472

[34] Case D, Darden T, T Cheatham I, Simmerling C, Wang J, et al.. (2008) AMBER 10. University of California, San Francisco.

[35] Case D, Cheatham T, Darden T, Gohlke H, Luo R, et al (2005) The Amber biomolecular simulation programs. J Comput Chem 26: 1668–1688. 16200636 10.1002/jcc.20290 PMC1989667

[36] Ponder J, Case D (2003) Force fields for Protein Simulations. In: Protein Simulations, 525 B Street, Suite 1900, San Diego, CA 92101–4495 USA: Academic Press Inc., volume 66 of Advances in Protein Chemistry. 27+. 10.1016/s0065-3233(03)66002-x 14631816

[37] Jorgensen WL, Chandrasekhar J, Madura JD, Impey RW, Klein ML (1983) Comparison of simple potential functions for simulating liquid water. J Chem Phys 79: 926–935.

[38] Ryckaert J, Ciccotti G, Berendsen H (1977) Numerical integration of the cartesian equations of motion of a system with constraints: Molecular dynamics of n-alkanes. J Comput Phys 23: 327–341.

[39] Berendsen H, Postma J, van Gunsteren W, DiNola A, Haak J (1984) Molecular dynamics with coupling to an external bath. J Chem Phys 81: 3684–3690.

[40] Senzaki H, Isoda T, Paolocci N, Ekelund U, Hare J, et al (2000) Improved mechanoenergetics and cardiac rest and reserve function of in vivo failing heart by calcium sensitizer EMD-57033. Circulation 101: 1040–1048. 10704173 10.1161/01.cir.101.9.1040

[41] Miller BR, McGee TD, Swails JM, Homeyer N, Gohlke H, et al (2012) MMPBSA.py: An efficient program for end-state free energy calculations. J Chem Theory Comput 8: 3314–3321. 26605738 10.1021/ct300418h

[42] Hou T, Wang J, Li Y (2011) WangW (2011) Assessing the performance of the MM/PBSA and MM/GBSA methods. 1. The accuracy of binding free energy calculations based on molecular dynamics simulations. J Chem Info Model 51: 69–82. 10.1021/ci100275a PMC3029230 21117705

[43] Moreira IS, Fernandes PA, Ramos MJ (2007) Computational alanine scanning mutagenesisan improved methodological approach. J Comput Chem 28: 644–654. 17195156 10.1002/jcc.20566

[44] Chong LT, Pitera JW, Swope WC, Pande VS (2009) Comparison of computational approaches for predicting the effects of missense mutations on p53 function. J Mol Graphics Model 27: 978–982. 10.1016/j.jmgm.2008.12.006 19168381

[45] Brice AR, Dominy BN (2011) Analyzing the robustness of the MM/PBSA free energy calculation method: Application to DNA conformational transitions. J Comput Chem 32: 1431–1440. 21284003 10.1002/jcc.21727

[46] Kollman PA, Massova I, Reyes C, Kuhn B, Huo S, et al (2000) Calculating structures and free energies of complex molecules: Combining molecular mechanics and continuum models. Acc Chem Res 33: 889–897. 11123888 10.1021/ar000033j

[47] Levy R, Zhang L, Gallicchio E, Felts A (2003) On the nonpolar hydration free energy of proteins: Surface area and continuum solvent models for the solute-solvent interaction energy. J Am Chem Soc 125: 9523–9530. 12889983 10.1021/ja029833a

[48] Singh N, Warshel A (2010) Absolute binding free energy calculations: On the accuracy of computational scoring of protein-ligand interactions. Proteins 78: 1705–1723. 20186976 10.1002/prot.22687 PMC2868600

[49] Barducci A, Bussi G, Parrinello M (2008) Well-tempered metadynamics: A smoothly converging and tunable free-energy method. Phys Rev Lett 100: 020603. 18232845 10.1103/PhysRevLett.100.020603

[50] Bonomi M, Branduardi D, Bussi G, Camilloni C, Provasi D, et al (2009) PLUMED: A portable plugin for free-energy calculations with molecular dynamics. Comput Phys Commun 180: 1961–1972.

[51] Dweck D, Reynaldo DP, Pinto JR, Potter JD (2010) A dilated cardiomyopathy troponin C mutation lowers contractile force by reducing strong myosin-actin binding. J Biol Chem 285: 17371–17379. 20371872 10.1074/jbc.M109.064105 PMC2878500

[52] Dong WJ, Xing J, Ouyang Y, An J, Cheung HC (2008) Structural kinetics of cardiac troponin C mutants linked to familial hypertrophic and dilated cardiomyopathy in troponin complexes. J Biol Chem 283: 3424–3432. 18063575 10.1074/jbc.M703822200

[53] Robinson P, Griffiths PJ, Watkins H, Redwood CS (2007) Dilated and hypertrophic cardiomyopathy mutations in troponin and alpha-tropomyosin have opposing effects on the calcium affinity of cardiac thin filaments. Circ Res 101: 1266–1273. 17932326 10.1161/CIRCRESAHA.107.156380

[54] Liu B, Tikunova SB, Kline KP, Siddiqui JK, Davis JP (2012) Disease-related cardiac troponins alter thin filament Ca2+ association and dissociation rates. PLoS ONE 7: e38259. 22675533 10.1371/journal.pone.0038259 PMC3366952

[55] Liou YM, Kuo SC, Hsieh SR (2008) Differential effects of a green tea-derived polyphenol (–)-epigallocatechin-3-gallate on the acidosis-induced decrease in the Ca2+ sensitivity of cardiac and skeletal muscle. Pugers Arch, EJP 456: 787–800. 10.1007/s00424-008-0456-y 18231806

[56] Lindhout D, Boyko R, Corson D, Li M, Sykes B (2005) The role of electrostatics in the interaction of the inhibitory region of troponin I with troponin C. Biochemistry. 44: 14750–14759. 10.1021/bi051580l 16274223

