# Correction: A Computational Exploration of the Interactions of the Green Tea Polyphenol (–)-Epigallocatechin 3-Gallate with Cardiac Muscle Troponin C

**DOI:** 10.1371/annotation/23964dab-0e50-4644-b3b1-7c9e4ee09958

**Published:** 2013-10-11

**Authors:** Dominic Botten, Giorgia Fugallo, Franca Fraternali, Carla Molteni

Errors were introduced during the production process for this manuscript. The Model columns in Tables 1 and 2 are incorrect. The correct versions of the Tables can be viewed here:

Table 1: 

**Figure:**
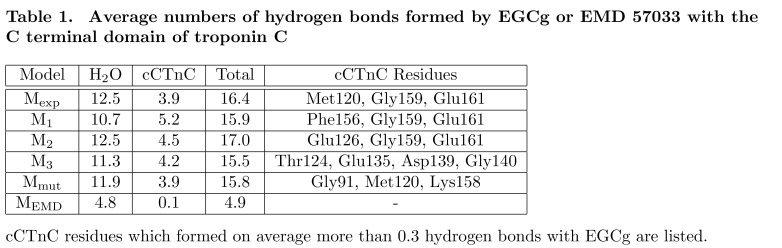


Table 2: 

**Figure:**
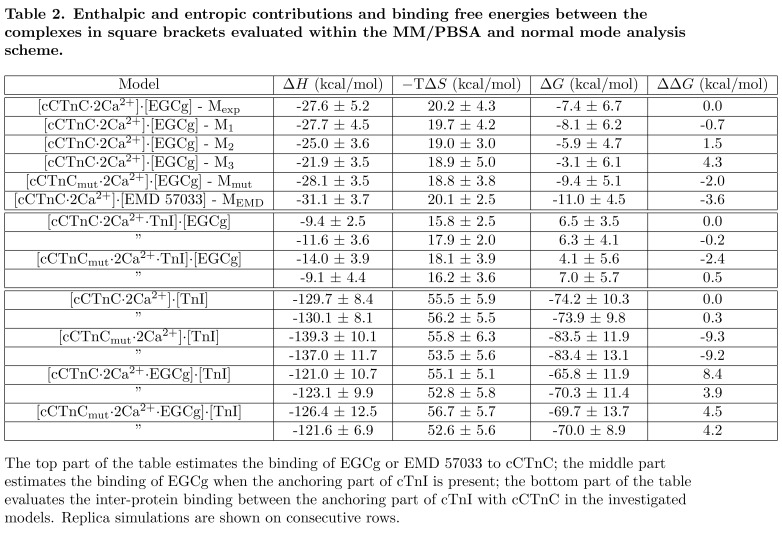


There are errors in References 3, 6, 7, and 8. The correct references are:

3. Nakayama M, Suzuki K, Toda M, Okubo S, Hara Y, et al. (1993) Inhibition of the infectivity of influenza virus by tea polyphenols. Antiviral Research 21: 289-299.

6. Hertog M, Feskens E, Hollman P, Katan M, Kromhout D (1992) Dietary antioxidant flavonoids and risk of coronary heart disease: the zutphen elderly study. Lancet 339: 1523-1526.

7. Riemersma R, Rice-Evans C, Tyrrell R, Cliord M, Lean M (2001) Tea flavonoids and cardiovasular health. Q J Med 94: 277.

8. Hodgson M (2008) Tea flavonoids and cardiovascular disease. Asia Pac J Clin Nutr 17: 288{290. 

